# Low expression of tRF‐Pro‐CGG predicts poor prognosis in pancreatic ductal adenocarcinoma

**DOI:** 10.1002/jcla.23742

**Published:** 2021-03-06

**Authors:** Jun Li, Lei Jin, Yuan Gao, Peng Gao, Le Ma, Bei Zhu, Xu Yin, Shizhen Sui, Shuai Chen, Zijian Jiang, Chunfu Zhu

**Affiliations:** ^1^ Dalian Medical University Dalian China; ^2^ Department of General Surgery The Affiliated Changzhou No. 2 People's Hospital of Nanjing Medical University Changzhou China

**Keywords:** fluorescence in situ hybridization, pancreatic ductal adenocarcinoma, tRF‐Pro‐CGG

## Abstract

**Background & Aims:**

tRFs (tRNA‐derived RNA fragments) have been reported to facilitate cancer progression in multiple cancers. However, their role in pancreatic ductal adenocarcinoma (PDAC) remains to be determined. In this study, we mainly investigated the expression of tRF‐Pro‐CGG in pancreatic ductal adenocarcinoma and evaluated its relationship with the clinicopathology and survival time of patients.

**Methods:**

37 cases of pancreatic ductal adenocarcinoma, and 15 cases of normal pancreatic tissues were collected which were resected by surgery from January 2017 to June 2020 from the Department of Hepatobiliary and Pancreatic surgery of Changzhou second people's Hospital. The expression of tRF‐Pro‐CGG in paraffin‐embedded tissues was detected by fluorescence in situ hybridization (FISH). The clinical data including age, sex, tumor location, tumor diameter, tumor clinical stage (TNM stage), depth of invasion, regional lymph node metastasis, serum CA199, and serum CEA were collected and analyzed retrospectively, whether the expression tRF‐Pro‐CGG was correlation with the pathological parameters and clinical outcomes of patients.

**Results:**

The expression level of tRF‐Pro‐CGG was significantly downregulated in PDAC and associated with an advanced TNM stage (P=0.000) and the N stage (P=0.000) of patients. More importantly, low tRF‐Pro‐CGG expression predicted poor survival in PDAC patients (P=0.003).

**Conclusions:**

TRF‐Pro‐CGG is under‐expressed in PDAC and is associated with short clinical survival and poor prognosis. tRF‐Pro‐CGG is an independent prognostic factor, which highlights its role as a potential biomarker for PDAC progression and therapy.

## Graphical abstract

Expression of tRF‐Pro‐CGG in pancreatic ductal adenocarcinoma and its clinical significance: Low expression of tRF‐Pro‐CGG predicts poor prognosis in pancreatic ductal adenocarcinoma. The expression of tRF‐Pro‐CGG has certain value in the diagnosis of PDAC patients.

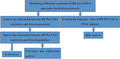

## 1 INTRODUCTION

In the European and American cancer statistics, PDAC is the fourth leading cause of cancer death.^1^, ^2^, ^3^ PDAC is one of the most aggressive gastrointestinal tumors with characteristics of early metastasis, local infiltration, and insensitivity to radiochemotherapy, and a 5‐year survival rate of only 9%.^1^, ^2^, ^3^ The treatment of pancreatic ductal adenocarcinoma generally includes early surgical resection and late chemotherapy. Due to early metastasis and chemotherapy tolerance, the effect of surgical resection and chemotherapy is not ideal, and most patients are not eligible for surgery after diagnosis.^3^ Thus, at present, the understanding of the pathogenesis, diagnosis, and treatment of pancreatic cancer needs to be further studied.

tRFs are small RNAs approximately 14–30 nt in length^4^, ^5^ that match the ends of mature and initial tRNA transcripts.^6^ tRFs are a type of tsRNAs, which share the specificity of tRNA in specific cells or tissues.^7^ tRFs are produced by specific cleavage under certain conditions by specific nucleases (dicer, ANG, etc.) or under circumstances of hypoxia or stress.^8^ According to the matched tRNA regions, tRFs can be divided into three categories, namely tRF‐5, tRF‐3, and tRF‐1.^6^, ^9^ tRF‐5 and tRF‐3 are derived from the 5′ and 3′ ends of mature tRNA, respectively, while tRF‐1 is derived from the 3′ end of the original tRNA. The length of tRF‐5 is approximately 1 to 30 nt, and it is formed by cutting the stem region and anticodon loop of the D loop or t loop of tRNA. The biological functions of tRFs are as follows: (1) tRFs can participate in the regulation of the expression of various genes.^10^ (2) tRFs can change the epigenetic status of genes.^11^, ^12^ (3) tRFs have a certain correlation with neuron degeneration.^13^ (4) tRFs can regulate chromatin.^14^ Since this type of tRF has a 5'‐phosphate and 3'‐OH and is similar in length to miRNA, it has miRNA‐like functions in cancer. For example, the biogenesis of dicer relies on tRFs to form RISC complexes with the argonaute protein and results from RNA silencing.^15^ Some miRNAs can be directly matched with tRFs, which inhibit the proliferation of tumor cells by binding to the YBX‐1 site.^16^, ^17^ In addition, under stress conditions, tRFs can promote the assembly of SGs, thereby inhibiting translation. According to relevant literature reports,^18^ tRFs play a role in the occurrence and metastasis of breast cancer,^16^ ovarian cancer,^19^ and prostate cancer,^20^ but no studies have been published regarding pancreatic ductal adenocarcinoma. Therefore, the expression levels and underlying mechanism of tRFs in PDAC remain to be elucidated.

Moreover, previously reported results showed that tRF‐Pro‐CGG (TRF‐001391), whose sequence is GAAGCGAGAA TCATACCCC T AGACCAACGA GCC, was differentially expressed in pancreatic cancer and normal pancreatic tissue based on tRF and tiRNA sequencing, which was verified by RT‐PCR in tissue.^21^ tRF‐Pro‐CGG was mostly enriched in “neuromuscular processes” (biological process), “neurons” (cellular component), and “PDZ domain binding” (molecular function) and was mostly enriched in “the PI3 K/protein kinase‐B signaling pathway.” However, the differential expression in pancreatic tissues and the clinical significance of tRF‐Pro‐CGG are still unclear.

The main purpose of this study was to verify the differential expression of tRF‐Pro‐CGG between pancreatic ductal adenocarcinoma tissue and normal patient pancreatic tissue and to examine the relationship between tRF‐Pro‐CGG expression and clinicopathological parameters. This may suggest that tRF‐Pro‐CGG is a potential tumor marker of PDAC.

## 2 MATERIALS AND METHODS

### 2.1 Ethics statement

Thirty‐seven samples of paraffin‐embedded human pancreatic ductal adenocarcinoma tissue, 15 samples of normal pancreatic tissue, and clinical‐pathological parameters were collected from 2017 to 2020 at our institute in accordance with the ethical standards of the institutional and/or national research committee and with the 1964 Declaration of Helsinki and its later amendments or comparable ethical standards. The research protocol was approved by the Ethics Committee of the Second People's Hospital of Changzhou City, Jiangsu Province, China. Paraffin tissue specimens were obtained with the patient's informed consent and written permission.

### 2.2 Patients

A total of 37 samples of paraffin‐embedded human pancreatic ductal adenocarcinoma tissue, 15 samples of normal pancreatic tissue, and clinical‐pathological parameters of Changzhou Second People's Hospital from January 2017 to June 2020 were collected. All specimens were confirmed to be pancreatic ductal adenocarcinoma by definitive histopathological diagnosis. Inclusion criteria were as follows: (1) ten percent formalin‐fixed and paraffin‐embedded (FFPE) pancreatic cancer tissue specimens; (2) pathologically confirmed pancreatic ductal adenocarcinoma; (3) complete clinical and pathological patient data; and (4) no radiotherapy or chemotherapy was administered before surgery. Exclusion criteria were as follows: (1) other primary tumors and (2) incomplete clinical and pathological data. The pathological diagnosis and TNM staging of pancreatic cancer tissue was based on the UICC/AJCC TNM staging system (8th edition, 2017).^22^ The thickness of each paraffin section was approximately 3 mm. Normal pancreatic tissue was obtained at least 2 cm from the edge of the pancreatic lesion and served as a control group.

### 2.3 Fluorescence in situ hybridization (FISH)

FISH was carried out on FFPE tissue sections according to the kit manufacturer's instructions (RNA FISH Kit; GenePharma). Briefly, the sections were deparaffinized in xylene and rehydrated with graded ethanol. The sections were then incubated with protease K and preheated deformation solution and hybridized with the probe. Finally, the sections were counterstained with Nuclear Blue using 4,6‐diamino‐2‐phenylindole (DAPI) after washing and observed under a fluorescence microscope (Olympus IX71‐DP73).

### 2.4 Evaluation of tRF‐Pro‐CGG expression

Two pathologists performed independent scoring without clinical data. The FISH results were based on the staining intensity score (0 points (negative), 1 point (weak positive) and 2 points (strong positive)) and according to the proportion of positive cells (0 points (0%), 1 point (scattered or 0%‐10% positive cells), 2 points (local distribution or 10%‐50% positive cells), and 3 points (diffuse distribution or more than 50% positive cells). The staining intensity score and the positive cell proportion score were added to obtain the FISH score, and PDAC tissues were divided into low‐expression and high‐expression groups. According to the FISH score, high expression in cells was defined as a score ≥2.

### 2.5 Statistical analysis

The data were analyzed with the software package SPSS, version 23.0. Fisher exact tests were used to assess associations between tRF‐Pro‐CGG expression and other parameters. Univariate survival analysis was performed with the Kaplan‐Meier method, and differences in survival curves were assessed with the log‐rank test. Multivariate survival analysis was performed using the Cox regression model. A *p* value <0.05 was considered statistically significant.

## 3 RESULTS

### 3.1 tRF‐Pro‐CGG was downregulated in PDAC

**FIGURE 1:**
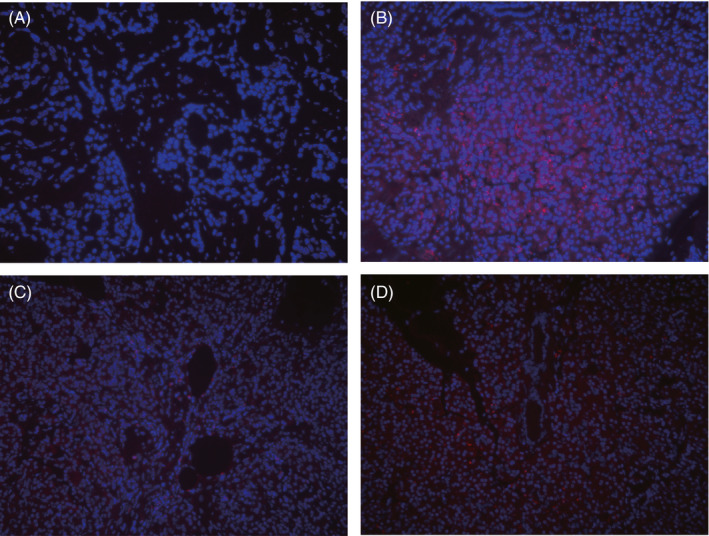
Localization of tRF‐Pro‐CGG expression in PDAC and normal pancreatic tissue by fluorescence in situ hybridization. A, Non‐expression of tRF‐Pro‐CGG in negative control group. B, Expression of tRF‐Pro‐CGG in normal pancreatic tissue. C, Low expression of tRF‐Pro‐CGG in the cancer nest of PDAC. D, High expression of tRF‐Pro‐CGG in the cancer nest of PDAC. Red, tRF‐Pro‐CGG; Blue, DAPI nuclear staining, 200×

**FIGURE 2:**
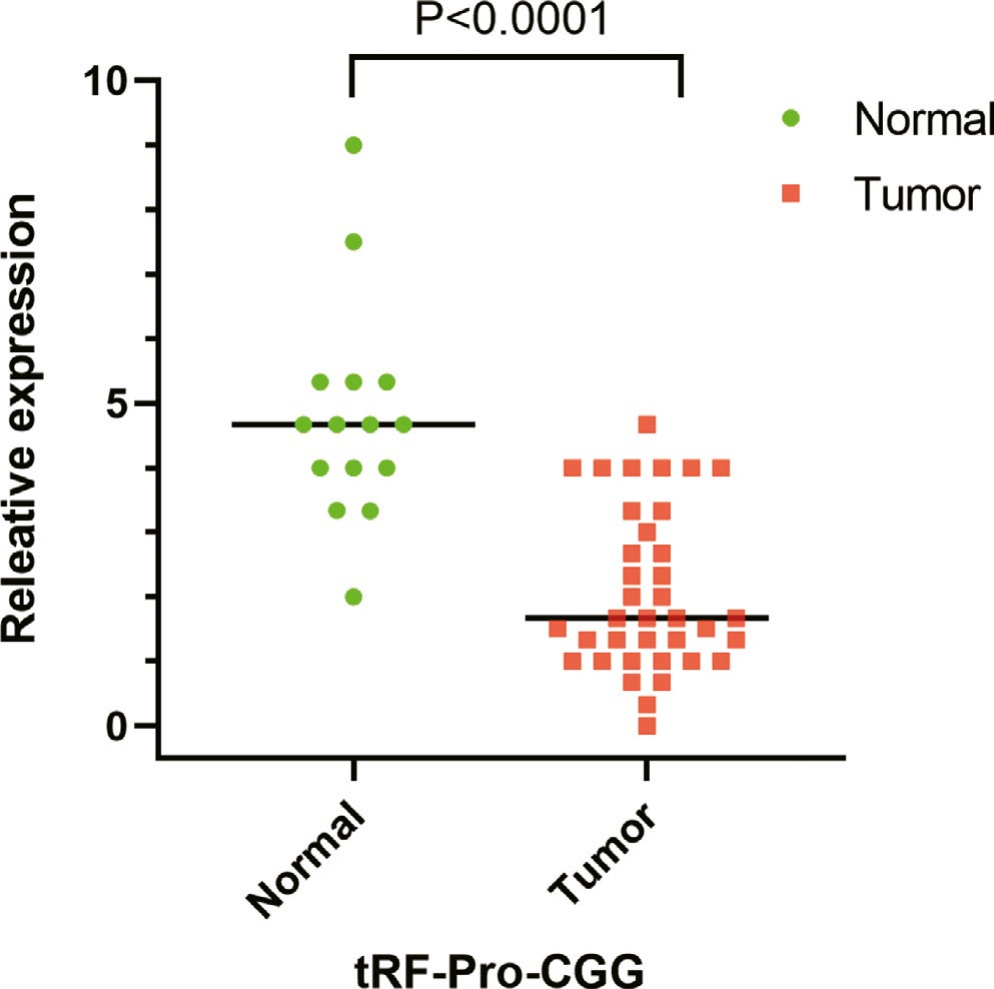
FISH detected the differential expression of tRF‐Pro‐CGG in pancreatic ductal carcinoma and normal pancreatic tissue

To explore tRF‐Pro‐CGG expression in PDAC, we performed FISH to detect tRF‐Pro‐CGG in FFPE pancreatic cancer tissue specimens. The results demonstrated that tRF‐Pro‐CGG was mainly expressed and localized in the cytoplasm of PDAC cells (Figure 1). Compared with the negative control sample (Figure 1A), certain normal pancreatic tissues had high tRF‐Pro‐CGG expression (Figure 1B). However, in cancerous tissues, tRF‐Pro‐CGG had low expression (Figure 1C,D). Compared with normal pancreatic samples, tRF‐Pro‐CGG was significantly downregulated in PDAC samples (Figure 2).

### 3.2 The expression level of tRF‐Pro‐CGG was associated with an advanced TNM stage

**FIGURE 3:**
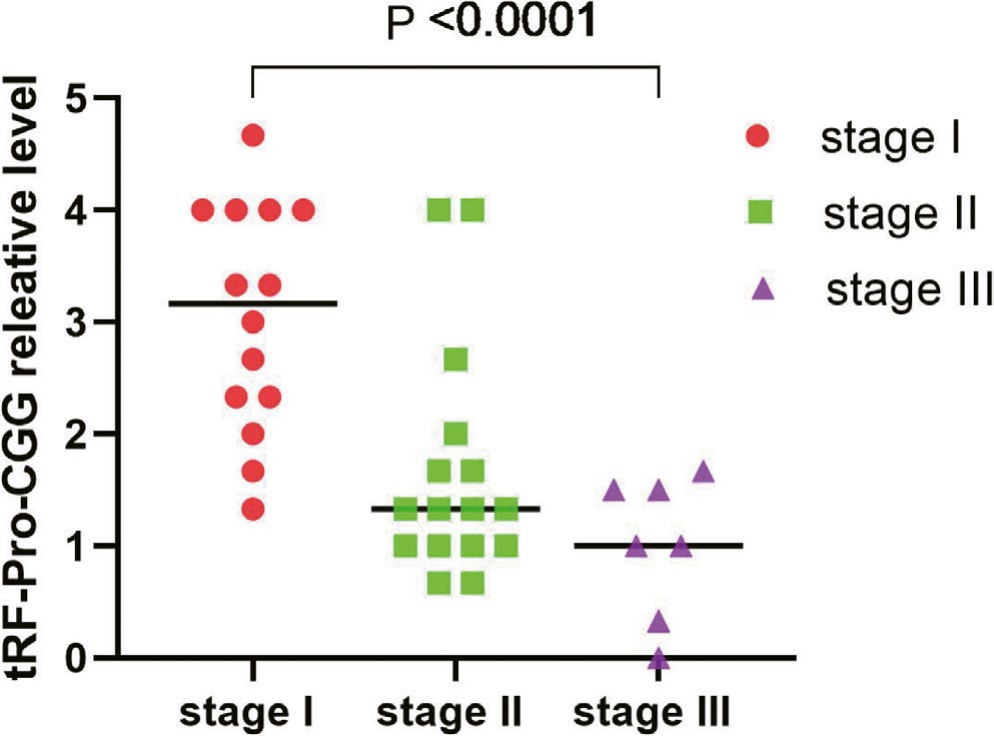
FISH detected the differential expression of tRF‐Pro‐CGG was grouped by the TNM stage

To explore the clinical function of tRF‐Pro‐CGG, we extracted all the pathological factors and conducted univariate analysis. The results are shown in Table 1. Among these pathological parameters, the expression difference in tRF‐Pro‐CGG was closely related to the TNM stage (*p* < 0.01). Further study revealed that lymphatic metastasis was a crucial indicator associated with high tRF‐Pro‐CGG expression in PDAC patients. According to the pathological stage, matched‐pair analysis demonstrated that tRF‐Pro‐CGG was significantly downregulated in advanced PDAC (Figure 3).

**TABLE 1:** Relationship between tRF‐Pro‐CGG expression and clinicopathological features of PDAC

Characteristics	Number of case	tRF‐Pro‐CGG expression		*p* value
		High group (n = 16)	Low group (n = 21)	
Age (years)				
≤60	12	3	9	0.166
>60	25	13	12	
Gender				
Male	22	11	11	0.5
Female	15	5	10	
TNM stage				
Ⅰ	14	12	2	0
Ⅱ‐Ⅲ	23	4	19	
Location				
Head	25	11	14	1
Tail	12	5	7	
N stage				
N0	18	14	4	0
N1‐N2	19	2	15	
T stage				
T1‐T2	26	13	13	0.285
T3‐T4	11	3	8	
Histological grade				
Well + Moderate	13	6	7	1
Poor	15	6	9	
Tumor size (cm)				
≤3	21	10	11	0.716
>3	16	6	10	
CEA				
≥5 ng/ml	10	5	5	1
<5 ng/ml	27	11	16	
CA199				
≤37 U/ml	9	4	5	1
>37 U/ml	28	12	16	

### 3.3 Low expression of tRF‐pro‐CGG predicts poor prognosis in PDAC patients

To further explore the clinical significance of tRF‐Pro‐CGG for predicting overall survival (OS), Kaplan‐Meier survival analysis was conducted to analyze the relationship between the outcome of patients and tRF‐Pro‐CGG expression. The results showed the overall survival (OS) curves for patients after curative surgery stratified by the tRF‐Pro‐CGG expression of the tumor. Patients with low tRF‐Pro‐CGG expression in the tumor had a significantly shorter OS (*p* = 0.003, log‐rank test) than patients with high tRF‐Pro‐CGG expression. The median OS was 7 months in the low‐expression group and 31 months in the high‐expression group.

To further identify prognostic factors from all the clinicopathological parameters, we performed variable factor analysis between survival time and clinicopathological parameters. The results demonstrated that patient survival time and clinicopathological characteristics were statistically similar between both cohorts with regard to age, sex distribution, location, CEA expression, and CA199 expression (Table 2). The expression of tRF‐Pro‐CGG (HR 6.09895 CI 1.969–18.881, *p* = 0.002), TNM stage (Ⅰ/Ⅱ‐Ⅲ) (HR 4.493, 95% CI 1.597–12.641, *p* = 0.004), and regional lymph node metastasis (N0/N1‐N2) (HR 3.822, 95% CI 1.470–9.937, *p* = 0.006) was associated with the prognosis of patients with PDAC.

**TABLE 2:** Univariate analysis of clinicopathological factors for disease‐specific survival in PDAC

Variable	PDAC (N)	HR (Hazard ratio)	95% CI	*p* Value
Age (years)				
≤60	12	1	0.330–1.185	0.555
>60	25	0.774		
Gender				
Male	22	0.961	0.417–2.298	0.961
Female	15	1		
TNM stage				
Ⅰ	14	1	1.597–12.641	0.004
Ⅱ‐Ⅲ	23	4.493		
Location				
Head	25	0.526	0.233–1.244	0.144
Tail	12	1		
N stage				
N0	18	1	1.470–9.937	0.006
N1‐N2	19	3.822		
T stage				
T1‐T2	26	1	0.698–4.048	0.247
T3‐T4	11	1.681		
Tumor size (cm)				
≤3	21	1	0.689–1.451	3.579
>3	16	1.57		
CEA				
≥5 ng/ml	10	1.04	0.419–2.582	0.933
<5 ng/ml	27	1		
CA199				
≤37 U/ml	9	1	0.323–2.469	0.828
>37 U/ml	28	0.894		
tRF‐Pro‐CGG				
Low	21	6.098	1.969–18.881	0.002
High	16	1		

**FIGURE 4:**
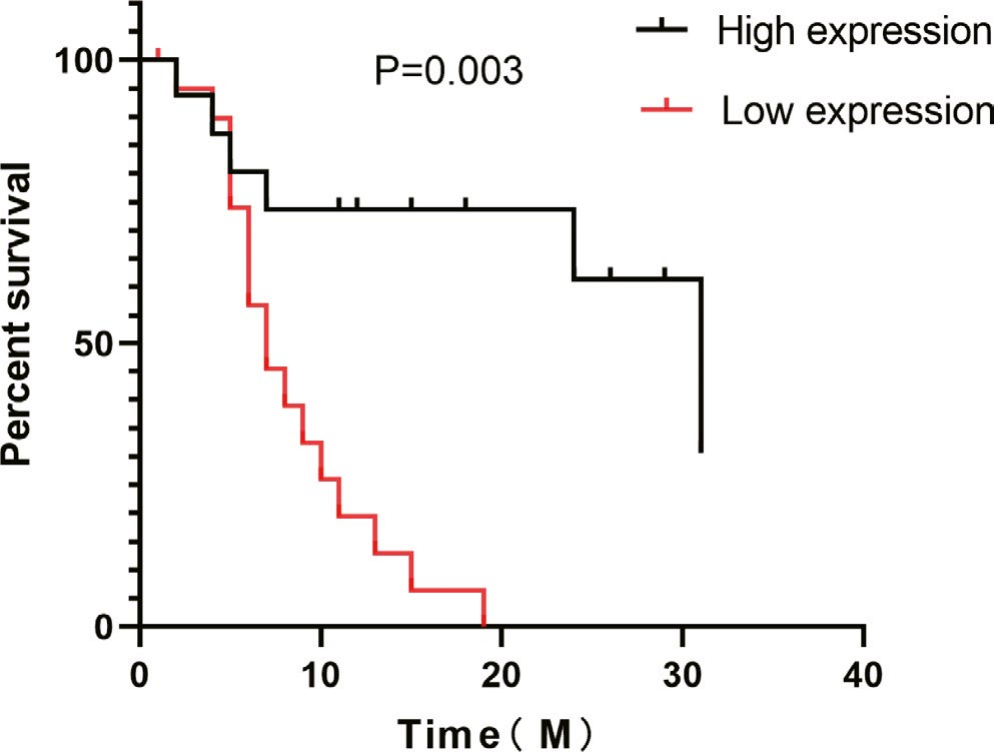
Kaplan‐Meier analysis comparing overall survival between the tRF‐Pro‐CGG high and low expression groups. Patients with low tRF‐Pro‐CGG expression had shorter overall survival (*p* = 0.003)

To further explore whether tRF‐Pro‐CGG can be used as one of the independent factors to predict the prognosis of patients, the indicators with statistical significance in the univariate such as analysis tRF‐Pro‐CGG expression, TNM stage (Ⅰ/Ⅱ‐Ⅲ), and regional lymph node metastasis (N0/N1‐N2) were included in COX regression model for multivariate analysis. The results demonstrated that the expression of tRF‐Pro‐CGG (HR 3.955,95% CI 1.053–14.853, *p* = 0.042) was correlated with the survival time of PDAC patients and was an independent risk factor for the prognosis of PDAC patients, while TNM stage (Ⅰ/Ⅱ‐Ⅲ) and regional lymph node metastasis (N0/N1‐N2) were not independent risk factors for the prognosis of PDAC patients. Therefore, the expression level of tRF‐Pro‐CGG can be used as a prognostic indicator of patients. Together, the data from Figure 4, Tables 2 and 3 suggest that the expression level of tRF‐Pro‐CGG can be used as a prognostic indicator of patients.

**TABLE 3:** Multivariate analysis of clinicopathological factors for disease‐specific survival in PDAC

Variable	HR	95% CI	*p* value
TNM stage	2.429	0.559–10.558	0.237
N stage	0.964	0.249–3.740	0.958
tRF‐Pro‐CGG	3.955	1.053–14.853	0.042

### 3.4 ROC analysis of the diagnostic value of tRF‐Pro‐CGG

**FIGURE 5:**
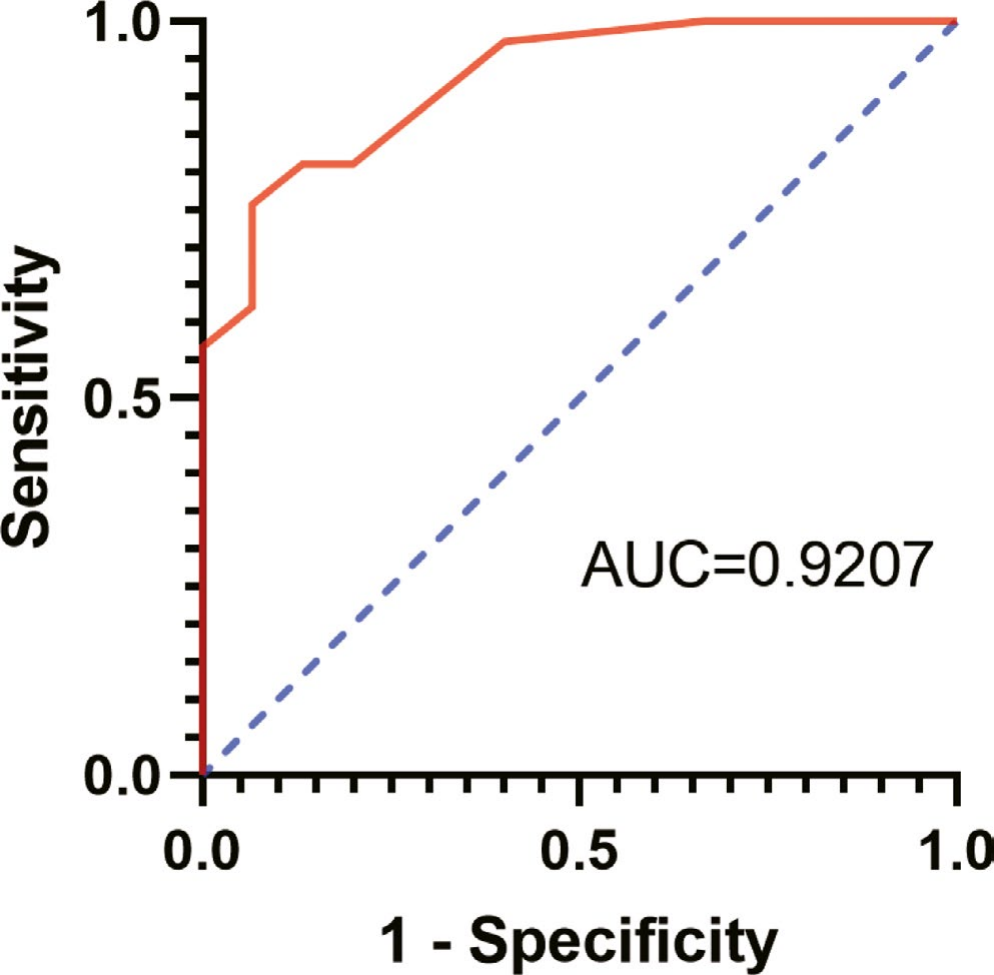
ROC curve to evaluate the diagnostic value of tRF‐Pro‐CGG in pancreatic ductal adenocarcinoma

Though tRF‐Pro‐CGG could act as an independent factor for predicting prognosis, its function in clinicopathologic diagnosis is still unknown. ROC analysis was used to evaluate the diagnostic value of tRF‐Pro‐CGG. The results shown in Figure 5 show that the AUC of tRF‐Pro‐CGG of the diagnostic curve was 0.92 (95% CI 0.8466–0.9948, *p* < 0.0001). The sensitivity and specificity were 75.7% and 93.3%, respectively.

## 4 DISCUSSION

Pancreatic cancer remains one of the malignancies with the worst prognosis despite recent advances in cancer treatment. Surgery is the only curative therapy. Combining surgery with adjuvant gemcitabine, which represents the current gold‐standard therapy for resected pancreatic cancer, improves outcomes, although with limited benefits.^23^ The characteristics of PDAC are local aggressiveness, early lymphatic and hematogenous dissemination, and chemotherapeutic resistance.^24^ The most common tissue diagnosis method is the fine‐needle aspiration (EUS‐FNA) sampling of pancreatic masses or other suspicious lesions guided by endoscopic ultrasonography (EUS).^25^ More than 80% of PDAC patients are already ineligible for surgical resection at diagnosis.^26^, ^27^ Currently, no tissue biomarkers are available to guide therapeutic strategies or predict patient prognosis in PDAC.

TRFs, small RNAs that are hydrolyzed fragments of tRNA have been increasingly reported to participate in multiple biological processes, such as RNA silencing,^28^ ribosomal biogenesis,^29^ long terminal repeat reverse transcriptional transposons,^30^ translational regulation,^31^, ^32^ viral infection,^33^, ^34^ and neurodegeneration. In addition, some sperm tRFs promote metabolism.^11^, ^12^ Obviously, tRFs play an important role in many cancers. However, there are no relevant reports about tRFs in PDAC. Thus, the role of tRFs in PDAC remains unclear.

Previously reported results showed that tRF‐Pro‐CGG (TRF‐001391), whose sequence is GAAGCGAGAA TCATACCCC T AGACCAACGA GCC, was differentially expressed in pancreatic cancer and normal pancreatic tissue by using tRF and tiRNA sequencing, which was verified by RT‐PCR in the tissue.^21^ To better analyze the role of tRF‐Pro‐CGG in PDAC, we evaluated tRF‐Pro‐CGG expression in pancreatic cancer using FISH and correlated tRF‐Pro‐CGG expression levels with the pathological parameters and clinical outcomes of patients after curative surgery. Interestingly, a large number of tRFs expressed in cancers are oncogenes, such as tRF‐1001 in prostate cancer,^20^ tRF‐25, tRF‐38, and tRF‐18 in gastric cancer,^35^ and tRF‐leu‐CAG in lung cancer.^36^ However, the results of this study showed that tRF‐Pro‐CGG was expressed at low levels in PDAC compared with normal pancreatic tissue, which was in agreement with the results of a previous study. Among the patients, 56.8% of patients with PDAC had relatively low expression. In addition, among these pathological parameters, the expression difference in tRF‐Pro‐CGG was closely related to the TNM stage (*p* < 0.01) and N stage (*p* < 0.01) of the tumor. The results suggest that the expression of tRF‐Pro‐CGG was related to the progression of pancreatic ductal adenocarcinoma, and tRF‐Pro‐CGG may be a tumor suppressor gene that inhibits the growth and metastasis of PDAC. This result is the same as the study that the expression of tRFs (5'‐tiRNA‐Val) is closely related to lymph node metastasis of colorectal cancer,^37^ but different from the correlation between tRFs/tiRNAs and clinicopathological parameters of patients with gastric cancer.^38^ In 2019, Zhu et al studied the relationship between tRFs (tiRNA‐5034‐GluTTC‐2) and clinicopathological parameters in 86 cases of gastric cancer. The results showed that the expression of tiRNA‐5034‐GluTTC‐2 was closely related to tumor diameter (*p* < 0.001), but not to lymph node metastasis and high stage.^37^ This shows that the expression of tRFs plays different roles in different cancers. In addition, the low expression of tRF‐Pro‐CGG gene in PDAC detected by FISH is closely related to the poor clinical outcome of these patients. The OS of patients with low expression of tRF‐Pro‐CGG in tumors (7 months) was significantly shorter than that of patients with high expression of tRF‐Pro‐CGG (31 months), which was consistent with the low expression of tRFs (tiRNA5034‐GluTTC‐2) in gastric cancer, and the correlation between low expression of tiRNA5034‐GluTTC‐2 and poor prognosis.^38^ This fully shows that the low expression of tRFs is related to poor prognosis. Univariate analysis showed that the low expression of tRF‐Pro‐CGG and clinicopathological factors such as TNM stage (Ⅰ/Ⅱ‐Ⅲ) and regional lymph node metastasis (N0/N1‐N2) were related to the prognosis of PDAC patients. Multivariate analysis showed that the low expression of tRF‐Pro‐CGG in tumor tissues was an independent risk factor for poor prognosis of patients with PDAC. The results of univariate analysis and multivariate analysis showed that the low expression of tRF‐Pro‐CGG may be an independent prognostic factor for patients with PDAC.

Finally, the receiver operating characteristic curve (ROC curve) was used to evaluate the diagnostic value of tRF‐Pro‐CGG in paraffin‐embedded tissues of PDAC. The AUC value of tRF‐Pro‐CGG diagnostic curve was 0.92% (95%CI 0.8466‐0.9948, *p* < 0.0001). The sensitivity is 75.7% and the specificity is 93.3%. The results show that the expression of tRF‐Pro‐CGG is related to the diagnosis of PDAC patients, and it is very likely to become a tumor biomarker for early diagnosis of PDAC patients. It is reported that more tRFs can be found in the exocrine than miRNA, which indicates that tRFs can be stably expressed in body fluids and rich in content. This makes them potential markers for early tumor diagnosis.^39^ In liver cancer, Zhu et al found that the level of plasma exocrine tRF‐5GluCTC in patients with liver cancer was significantly higher than that in healthy controls, suggesting that tRFs in plasma exosome can be used as a biomarker for early diagnosis of liver cancer.^40^ In gastric cancer, the receiver working curve of Zhang et al showed that tRF‐3019a could distinguish tumor tissue from non‐tumor tissue, and its AUC was 0.689. Although the diagnostic potential of tRF‐3019a is not ideal, it is still better than the previously reported values of CEA (AUC = 0.583) and CA199 (AUC = 0.585). TRF‐3019a can be used as a suitable biomarker for (GC) diagnosis of gastric cancer.^41^ Dong et al found that the ROC curve AUC of tRF‐24‐V29K9UV3 IU expression level was 0. 8712 (95% CI 77.35% ‐ 98.73%). The sensitivity and specificity of tRF‐24‐V29K9UV3 IU expression in GC were 78.57% and 92.86%, respectively. TRF‐24‐V29K9UV3 IU can be used as a biomarker for the diagnosis of gastric cancer.^42^ In short, tRFs may become one of the tumor biomarkers for early diagnosis of malignant tumors.

The 5‐year survival rate of PDAC patients treated with either surgery or neoadjuvant chemoradiotherapy is less than 30% despite complete resection.^26^, ^43^ The results show for the first time that tRF‐Pro‐CGG expression in FFPE samples can be used to delineate a subgroup of patients who will truly benefit from subsequent surgery. Patients whose cancers express high levels of tRF‐Pro‐CGG are predicted to have a greater than 50% survival rate after two years. In contrast, low levels of tRF‐Pro‐CGG predict poor outcomes and short OS after surgical resection.

In recent years, research on tumor growth and the migratory and invasive abilities of cancers, including PDAC, has increased. However, the mechanisms underlying tRF‐mediated aggression in PDAC remain unclear. Furthermore, the role of tRFs in suppressing tumor proliferation and invasion among different types of tumors is still disputed. For example, recent studies have shown that tRFs are decreased in breast cancer and B cell lymphoma and suppress cell proliferation by competitively binding to YNBX or its analogues^16^ or suppressing the expression of endogenous RAP1.^14^ However, research has also shown that tRFs can increase cell proliferation and migration by promoting cell cycle progression^20^ or can enhance the proliferation of lung cancer cells by regulating AURKA.^36^ The significant difference in the expression of tRFs in various tumors may cause them to play different roles. In addition, tRFs can interact with the promoter regions of a variety of genes to cause the downregulation of the expression of genes in early embryos, indicating that tRF expression is associated with epigenetic modifications in promoters.^11^ In addition, Telonis et al. found that tRFs were associated with the regulation of the transfer‐related MAPK and wnt/β‐catenin signaling pathways, which is consistent with the results of our previous study. Thus, the role of tRF‐Pro‐CGG and its regulatory mechanism in pancreatic cancer cell lines need to be further studied.

In this study, we used FISH to evaluate the expression intensity and distribution of tRF‐Pro‐CGG in pancreatic cancer FFPE tissue. Fluorescence in situ hybridization (FISH) is a standard technique that provides information for routine diagnostics of aberrations. It has advantages of sensitivity, a strong signal, low background, and speed, and FISH probes have been used in conventional diagnostic methods for different types of cancer. However, IHC is now widely used in pathology to determine gene expression in PDAC tissue. It has been reported in the literature that IHC may overestimate positivity and increase the false‐positive rate of cells.^44^ Meanwhile, IHC detects the overexpression of receptor proteins, while FISH detects the amplification level of genes, especially small molecule gene fragments. FISH can also determine where genes are located in cells. It is faster, easier, and more widely available than PCR. Thus, FISH was used in this study.

This study had some limitations that should be considered. First, this study was retrospective and non‐random. We need to perform many prospective studies to further verify the reliability of the results. Second, the small number of patients with PDAC and the lack of adequate normal pancreatic tissue as a control group made the study less reproducible. We need large numbers of patients and multi‐center collaborations. Finally, we were unable to obtain sufficient complete patient clinical data for subgroup analysis.

## 5 CONCLUSION

In summary, this study showed that tRF‐Pro‐CGG is under‐expressed in PDAC and is associated with poor prognosis. tRF‐Pro‐CGG was an independent prognostic factor, which highlights its role as a potential biomarker for PDAC progression and therapy.

## CONFLICT OF INTEREST

The authors declare that this research is not related to any commercial or financial interests.

## AUTHOR CONTRIBUTIONS

Chunfu Zhu and Lei Jin conceived and designed the experiment. Jun Li and Yuan Gao wrote and revised the original article. Shizhen Sui and Xu Yin collected patient information and postoperative follow‐up. Peng Gao and Shuai Chen collected patient specimens, and Jun Li and Bei Zhu performed statistical analysis.

## Data Availability

The datasets used and/or analyzed during the current study are available from the corresponding author on reasonable request.
